# Analysis of *PIK3CA* mutations in the lysate of sentinel lymph nodes in patients with early breast cancer

**DOI:** 10.3389/fonc.2026.1658786

**Published:** 2026-03-09

**Authors:** Sung Ae Park, Nanae Masunaga, Takanori Kin, Ryu Tokui, Yasufumi Sato, Chieko Mishima, Tetsuhiro Yoshinami, Masami Tsukabe, Yoshiaki Sota, Tomonori Tanei, Kenzo Shimazu

**Affiliations:** 1 Department of Breast and Endocrine Surgery, Osaka International Cancer Institute, Osaka, Japan; 2 Department of Breast and Endocrine Surgery, The University of Osaka Graduate School of Medicine, Osaka, Japan

**Keywords:** breast cancer, droplet digital PCR, one-step nucleic acid amplification, PIK3CA, sentinel lymph node

## Abstract

**Background:**

Although one-step nucleic acid amplification (OSNA), which measures cytokeratin (CK) 19 mRNA copies, is used for intraoperative detection of sentinel lymph node (SN) metastasis, CK19 mRNA copy number may not always accurately reflect total tumor load in the SN. Because number of DNA copies per cell generally has smaller deviation, we hypothesized that detection of tumor-derived mutated DNA in SNs, by targeting genetic mutations in the primary tumor, may provide more accurate results than OSNA. We investigated the *PIK3CA* mutation, frequently detected in breast cancer, to explore the potential of this technique for diagnosing SN metastasis.

**Methods:**

We analyzed data from 94 patients who had undergone SN biopsy at Osaka University Hospital (April 2017 to March 2019). Next-generation sequencing was used for mutation analysis of the primary tumor. In cases of *PIK3CA* mutations, OSNA lysates were analyzed to detect *PIK3CA* mutations in the SN by using droplet digital polymerase chain reaction (ddPCR).

**Results:**

*PIK3CA* mutations were detected in 33.0% (31/94) of primary tumors, 25 of which had hotspot *PIK3CA* mutations and included 59 SNs. Of these SNs, 10 were diagnosed as metastasis positive by OSNA and confirmed by ddPCR to have *PIK3CA* mutations, with no false negatives.

**Conclusions:**

Assessment of tumor-derived mutated DNA in SNs may be a useful technique to detect SN metastasis, as confirmed by ddPCR analysis. Further analyses, using data from a greater number of patients, are necessary to determine whether the results of whole-genome and whole-exome sequencing can be applied to other genes.

## 1 Introduction

Sentinel lymph node biopsy (SLNB) is commonly performed intraoperatively to diagnose axillary lymph node metastasis (1–3). One-step nucleic acid amplification (OSNA) is used for this purpose (4, 5); it entails measuring amplification of cytokeratin (CK)19 mRNA in sentinel lymph nodes (SNs) to determine the presence of metastasis and has the same accuracy as histopathological examination (6). Advantages of OSNA include the facts that it assesses the whole node and that results can be obtained without the need for the involvement of a pathologist.

The results of the ACOSOG Z0011 trial indicated that, under certain conditions, axillary lymph node dissection (ALND) can be omitted even for patients with axillary lymph node metastases at the time of SLNB (7). However, the omission of ALND makes accurate assessment of axillary lymph node metastases difficult. Therefore, the sum of CK19 mRNA in the SN has been evaluated as total tumor load (TTL), which has been reported to be useful in predicting non-SN metastasis, and patient prognosis (8–11). However, TTL determination by OSNA lacks sensitivity in the prediction of total number of tumor cells in the SN, because copy numbers of CK19 mRNA differ greatly; in fact, a 30-fold variation in CK19 mRNA copy number has been reported (6).

Previous studies have shown that DNA per tumor cell is less variable than mRNA (12). By measuring the copy number of tumor-derived DNA and identifying whole tumor cells, we expect to be able to more accurately assess the metastatic status of axillary lymph nodes. We have previously developed and reported the potential of the restriction enzyme–based digital methylation−specific polymerase chain reaction (RE-dMSP) assay to evaluate tumor-derived DNA; this method targets the methylated *RASSF1A* gene expressed in breast cancer cells (12). However, because methylation negativity is found in approximately 10%–20% of all breast cancers (13, 14), the application of the RE-dMSP method is limited to primary tumor methylation positivity. Therefore, we propose the possibility of diagnosis of SN by targeting case-specific mutated genes that can be applied to all breast cancers (15, 16), and we are investigating its accuracy in the diagnosis of metastasis.

We carried out the present study to analyze mutated genes in SNs. We targeted *PIK3CA*, which is mutated in 40% of all breast cancers (17), as a gene mutation to be analyzed in lysates. Droplet digital polymerase chain reaction (ddPCR) was used to analyze H1047R, E545K, and E542K of *PIK3CA* in lysates; these have been identified as hotspots (17). Genes related to the PI3K/AKT signaling pathway, including *AKT1* and *PTEN*, were also analyzed.

## 2 Patients and methods

### 2.1 Patients and samples

**Figure 1:**
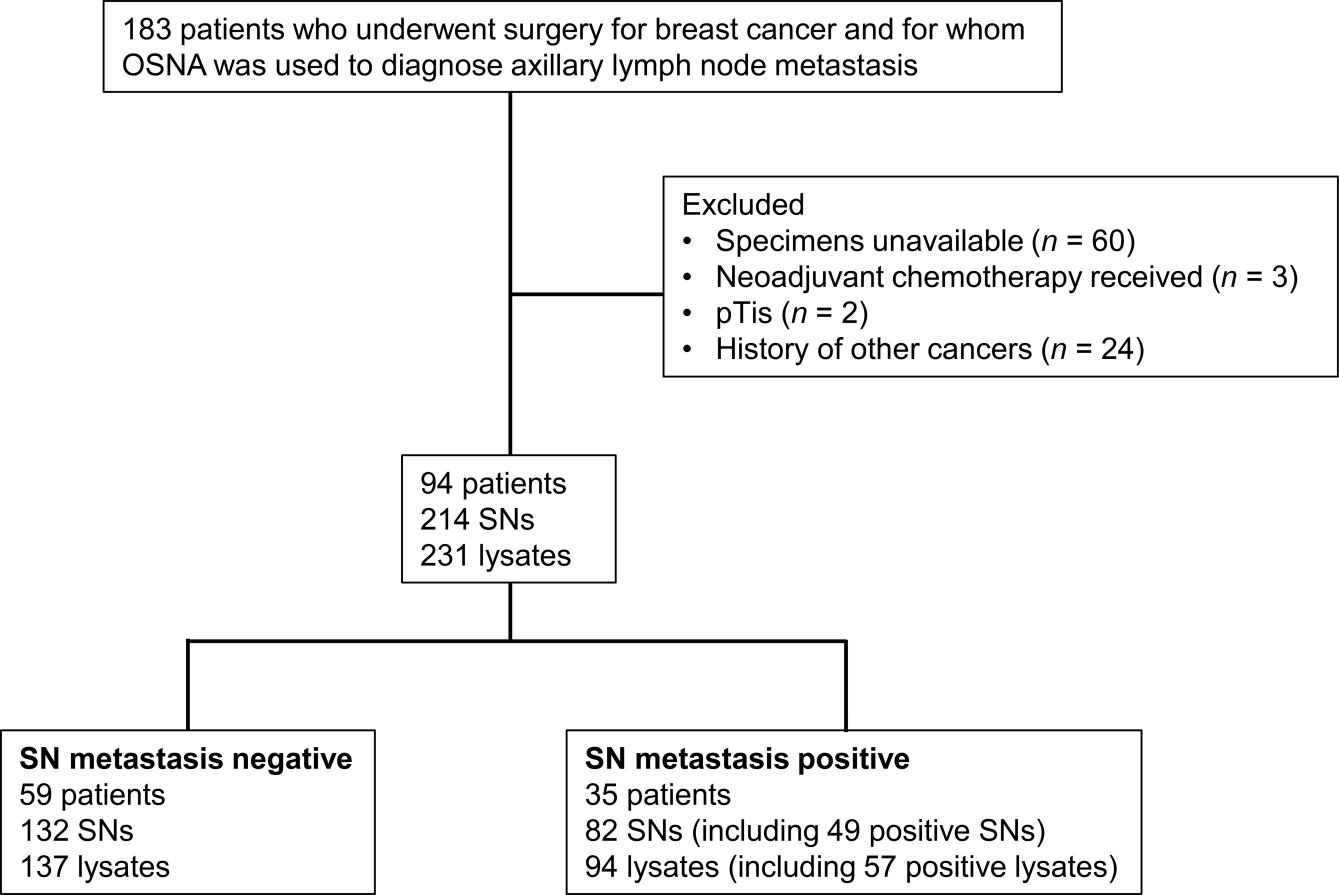
Patient selection process used in the present study. OSNA, one-step nucleic acid amplification; SN, sentinel node.

This retrospective study began with a review of data from 183 breast cancer patients who underwent surgery with SLNB, and whose SNs were examined by OSNA at Osaka University Hospital between April 2017 and March 2019. After applying the exclusion criteria, data from a total of 94 patients were ultimately used in the analysis of mutations in the primary tumor (**Figure 1**).

Tumors were obtained at the time of surgery and immediately frozen and stored at –80 °C until analysis by next-generation sequencing. SLNB was performed using both dye (patent blue or indocyanine green) and radiocolloid (technetium-99m tin colloid). The whole SN tissue was used for OSNA and homogenized in 4 mL of Lynorhag solution (Sysmex Corporation, Kobe, Japan); 2 μL of lysate was used for the assay, and the remainder was stored at –80 °C. To ensure adequate homogenization, large lymph nodes were divided into multiple tissue fragments for processing; thus, a single node could generate more than one lysate.

The classification of CK19 mRNA copy number per assay was as follows: >5000, (++); >250 and ≤5000, (+); >0 and ≤250 (–), and 0, (ND). As recommended by the manufacturer of the OSNA assay, (++) and (+) were considered positive for SN metastasis, and >0 and ≤250 were regarded negative for SN metastasis, despite the amplification of CK19 mRNA. In cases of a positive OSNA result, the decision as to whether or not to perform ALND was based on a nomogram used to predict non-SN metastasis (10, 18). This nomogram was developed at our institution to guide decision making regarding ALND, and its use has been investigated through a clinical trial approved by the Institutional Review Board of Osaka University Hospital (approval no. 21433).

**Figure 2:**
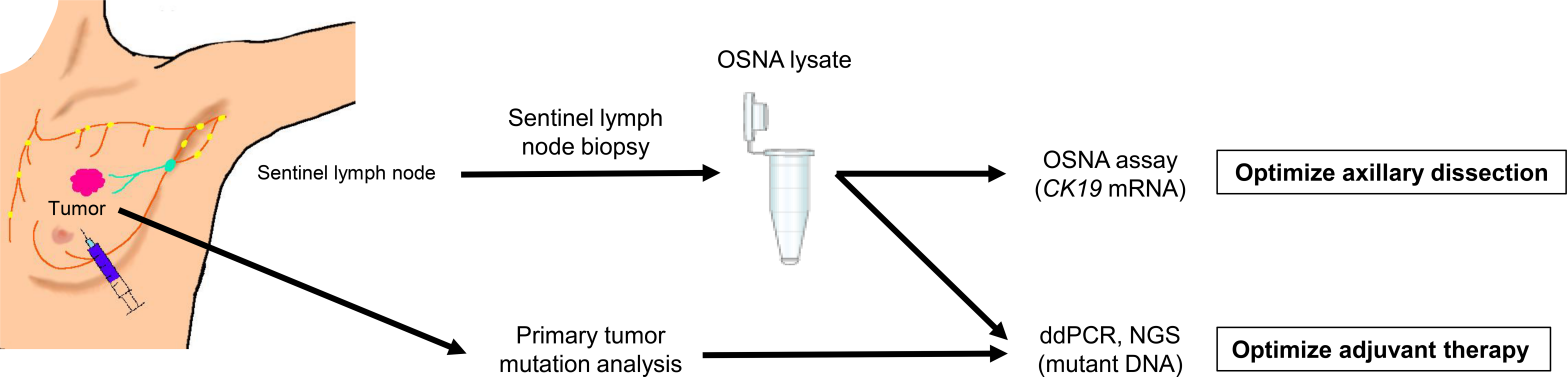
Detection of *PIK3CA* mutations in the present study, using one-step nucleic acid amplification (OSNA) lysates obtained from patients with breast cancer. ddPCR, droplet digital polymerase chain reaction; NGS, next-generation sequencing.

A total of 214 SNs and 231 lysates from 94 patients were analyzed. Mutation analysis of 21 genes in primary tumors was performed by next-generation sequencing in all cases. For cases of *PIK3CA* mutations in the primary tumor, *PIK3CA* hotspot mutations (*PIK3CA* H1047R, E545K, and E542K) in SN lysates were analyzed using ddPCR (**Figure 2**). The PI3K/AKT signaling pathway–related genes (*AKT1* and *PTEN*) were analyzed using molecular barcode–based next-generation sequencing. The detailed protocol followed that described by Yoshinami et al. (19).

### 2.2 DNA extraction

Tumor DNA was extracted from fresh frozen tissue using the DNeasy Blood & Tissue Kit (Qiagen, Hilden, Germany), according to manufacturer instructions. Peripheral blood lymphocytes were separated from whole blood by centrifugation for 10 min at 3000 rpm (1840 × g) twice and stored in the pellet state at –80 °C until further use. Peripheral blood lymphocyte DNA was extracted from the pellet using the DNeasy Blood & Tissue Kit, according to manufacturer instructions. Sentinel lymph node DNA was extracted from 100–500 μL of OSNA lysate, using the QIAamp Circulating Nucleic Acid Kit (Qiagen), and eluted using 50 μL of desalted water.

### 2.3 Primary tumor mutation analysis

The gene panel used in this study was an institutionally designed custom target panel generated using Agilent’s SureDesign platform (https://earray.chem.agilent.com/suredesign). The panel was not a commercially supplied next-generation sequencing kit but was specifically designed for this study to cover the whole exonic regions of 21 genes, including 12 genes frequently mutated in breast cancer (cBioPortal for Cancer Genomics, http://www.cbioportal.org) as well as *ESR1* (**Supplementary Table 1**). The median coverage of the sequencing area was 100% (range, 92%–100%) of the whole exome for each gene. Sequence libraries were prepared from 200 ng of tumor DNA, using a SureSelect XT HS2 (Agilent Technologies, Inc., Santa Clara, CA, USA) according to manufacturer instructions, and sequenced using MGI DNBSEQ-G400RS (MGI Tech, Shenzhen, China). CLC Genomics Workbench (Qiagen) was used for variant calling.

The exclusion criteria were as follows: variant allele frequency, <5%; read depth, <100; and forward–reverse balance, <0.2, in introns, reported as single nucleotide polymorphism with variant allele frequency >0.5% at the Tohoku Medical Megabank Organization (https://www.megabank.tohoku.ac.jp/english/).

### 2.4 Detection of *PIK3CA* mutation in SNs, using ddPCR

For mutation analysis, DNA was extracted from SN lysates as described above. Then, 9 μL of DNA solution was made up to 20 μL by mixing with the following solutions: 1XddPCR Supermix for probes (Bio-Rad Laboratories, Inc., Hercules, CA, USA), 900 nM for each primer, 250 nM for probe, and DNA. The sequences of the primers and probes are summarized in **Supplementary Table 2**.

Droplet generation oil was added to the mixture, which was subsequently transferred onto a QX100 droplet generator (Bio-Rad Laboratories, Inc.). Then, 40 μL of emulsified mixture was subjected to polymerase chain reaction using a T100 thermal cycler (Bio-Rad Laboratories, Inc.) at 95 °C for 10 min, followed by 40 cycles at 94 °C for 30 s, and 56 or 60 °C for 1 min, and 98 °C for 10 min.

Data analysis was performed using the QX100 droplet reader and QuantaSoft software, version 1.7.4 (both Bio-Rad Laboratories, Inc.). The presence of two or more dots per well was considered a positive result, and the copy numbers of three positive wells were summated. For cases divided into multiple lysates, the node was defined as positive if at least one lysate was ddPCR-positive.

### 2.5 Statistical analysis

R version 4.3.3 was used for statistical processing. Pearson’s correlation analysis was used to evaluate the association between copy numbers of mutated DNA and CK19 mRNA in lysates. Because both variables showed right-skewed distributions, log-transformed values were used. A *p* value < 0.05 was considered significant.

### 2.6 Ethics statement

The study was approved by the Ethical Review Board of Osaka University Hospital (approval date and number: January 29, 2019, #18396). Before surgery, all patients provided opt-out consent for the initial storage of samples and the use of samples in this study.

## 3 Results

### 3.1 Analysis of mutated genes in primary tumors

**Figure 3:**
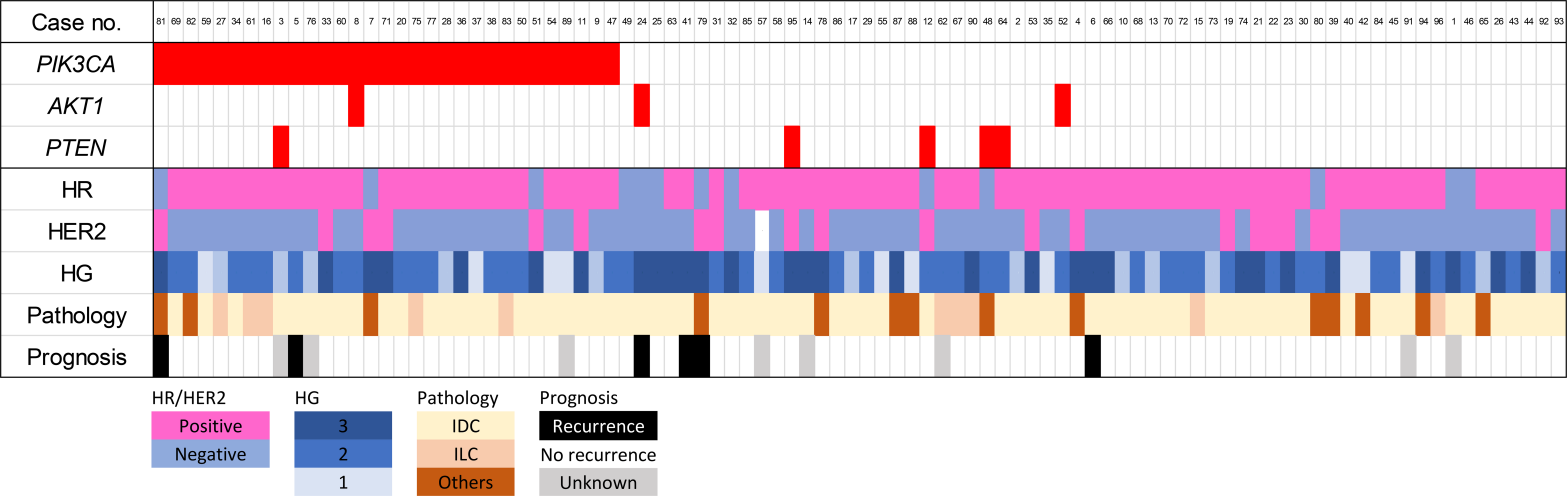
Clinicopathological information for patients in the present study. HER2, human epidermal growth factor receptor 2; HG, histological grade; HR, hormone receptor; IDC, invasive ductal carcinoma; ILC, invasive lobular carcinoma.

Data from 94 patients were evaluated (**Figure 1**), and the median follow-up period was 78.0 months (range, 1–105 months). Their clinicopathological characteristics are summarized in **Table 1**, **Figure 3**.

**Table 1:** Clinicopathological characteristics of the 94 patients with breast cancer whose data were used in the present study.

Characteristic	*n*
Age, years	
< 50	30
≥ 50	64
SN metastasis	
Negative	59
Positive	35
Axillary lymph node dissection	
No	67
Yes	27
Histological diagnosis	
Invasive ductal carcinoma	70
Invasive lobular carcinoma	10
Other^a^	14
Tumor size, mm	
< 20	42
≥ 20	52
Grade	
1 or 2	64
3	30
Lymphatic vessel invasion	
Positive	16
Negative	78
Subtype	
HR positive^b^, HER2 negative	66
HER2 positive	20
Triple negative	7
Unknown	1

HER2, human epidermal growth factor receptor 2; HR, hormone receptor; SN, sentinel lymph node.

a Apocrine (*n* = 6), micropapillary (*n* = 4), mucinous (*n* = 3), invasive carcinoma with neuroendocrine differentiation (*n* = 1).

b Either estrogen or progesterone receptor positive.

We performed panel sequencing of 21 genes in 94 patients and found mutations in 63 primary tumors (67.0% of patients). A total of 16 genes were mutated: *PIK3CA* in 31 cases (33.0%), *PTEN* in 5 cases (5.3%; one case harbored two additional *PTEN* mutations), and *AKT1* in 3 cases (3.2%) (**Supplementary Table 1**). In the 31 patients with *PIK3CA* mutations, mutations in hotspots H1047R, E545K, and E542K were detected in 13 (41.9%), 7 (22.6%), and 5 (16.1%), respectively (**Supplementary Table 3**). The breast cancer subtypes in these 31 patients were as follows: HR-positive and HER2-negative (*n* = 25), HER2-positive (*n* = 6); none had triple-negative breast cancer. Two patients with *PIK3CA* mutation–positive primary tumors developed recurrence during follow-up; the mutation was not a hotspot in either case.

### 3.2 Detection of PI3K/AKT signaling pathway-related gene (*PIK3CA*, *AKT1*, and *PTEN*) mutations in OSNA lysates

**Figure 4:**
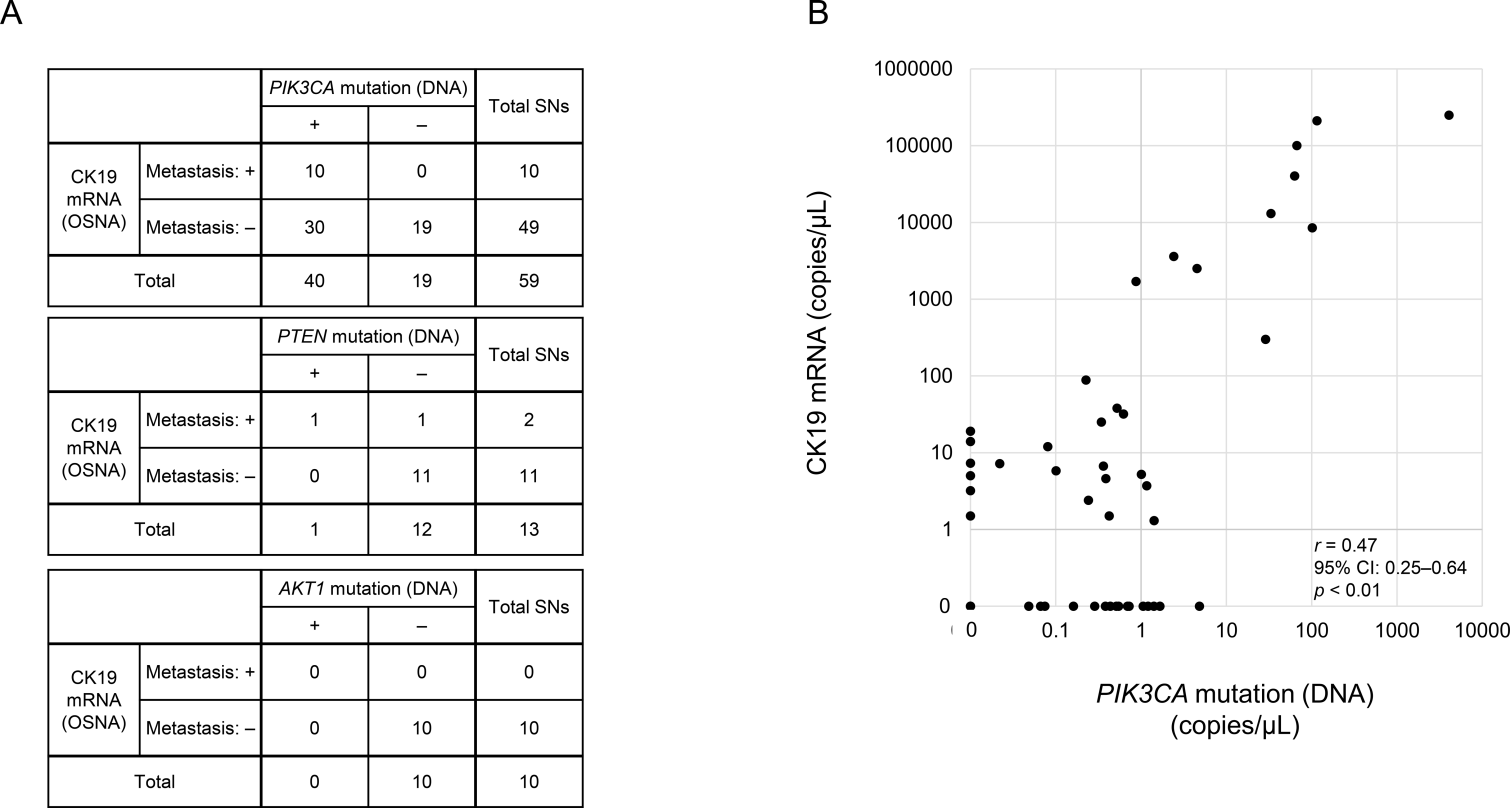
Correlations between CK19 mRNA and PI3K/AKT signaling pathway–related gene mutations in one-step nucleic acid amplification (OSNA) lysates. **(A)** Numbers of each mutant DNA-positive and negative sentinel nodes (SNs), stratified by OSNA diagnosis. **(B)** Correlation between CK19 mRNA and *PIK3CA* mutations in OSNA lysate DNA, in terms of copy number (copies/assay), determined by OSNA and droplet digital polymerase chain reaction, respectively. Pearson’s correlation analysis was used to assess the association, and a *p* value < 0.05 was considered statistically significant. CI, confidence interval.

We analyzed 59 SNs and 66 lysates in 25 patients with *PIK3CA* mutations: 1 SN, 2 SNs, and ≥3 SNs from each of 6, 9, and 10 patients, respectively. Intraoperative OSNA diagnosis of the 59 SNs showed that 10 (from 7 patients) were metastasis positive; 6 SNs (from 4 patients) were (++) and 4 (from 3 patients) were (+). In all 10 SNs determined metastasis positive by the OSNA, *PIK3CA* mutations were found. However, *PIK3CA* mutations were also detected in 30 of the 49 SNs (equivalent to 18 of 25 patients) diagnosed as negative for metastasis by the intraoperative OSNA (**Figure 4A**). For the five patients with *PTEN* mutations, 13 SNs and corresponding 13 lysates were analyzed. Intraoperative OSNA examination identified 2 of the 13 SNs (equivalent to 2 of 5 patients) as metastasis-positive; both cases were classified as micrometastasis. *PTEN* mutations were detected in only one SN, which was one of the metastasis-positive nodes. For the three patients with *AKT1* mutations, 10 SNs and the corresponding 10 lysates were analyzed. Intraoperative OSNA examination showed that none of the 10 SNs were metastasis-positive, and no *AKT1* mutations were detected in any SN. Because *PTEN* and *AKT1* mutations were only rarely detected in OSNA lysates using molecular barcode–based next-generation sequencing, we focused on *PIK3CA* for subsequent analysis.

### 3.3 Association between *PIK3CA* mutations and CK19 mRNA in OSNA lysates

**Figure 5:**
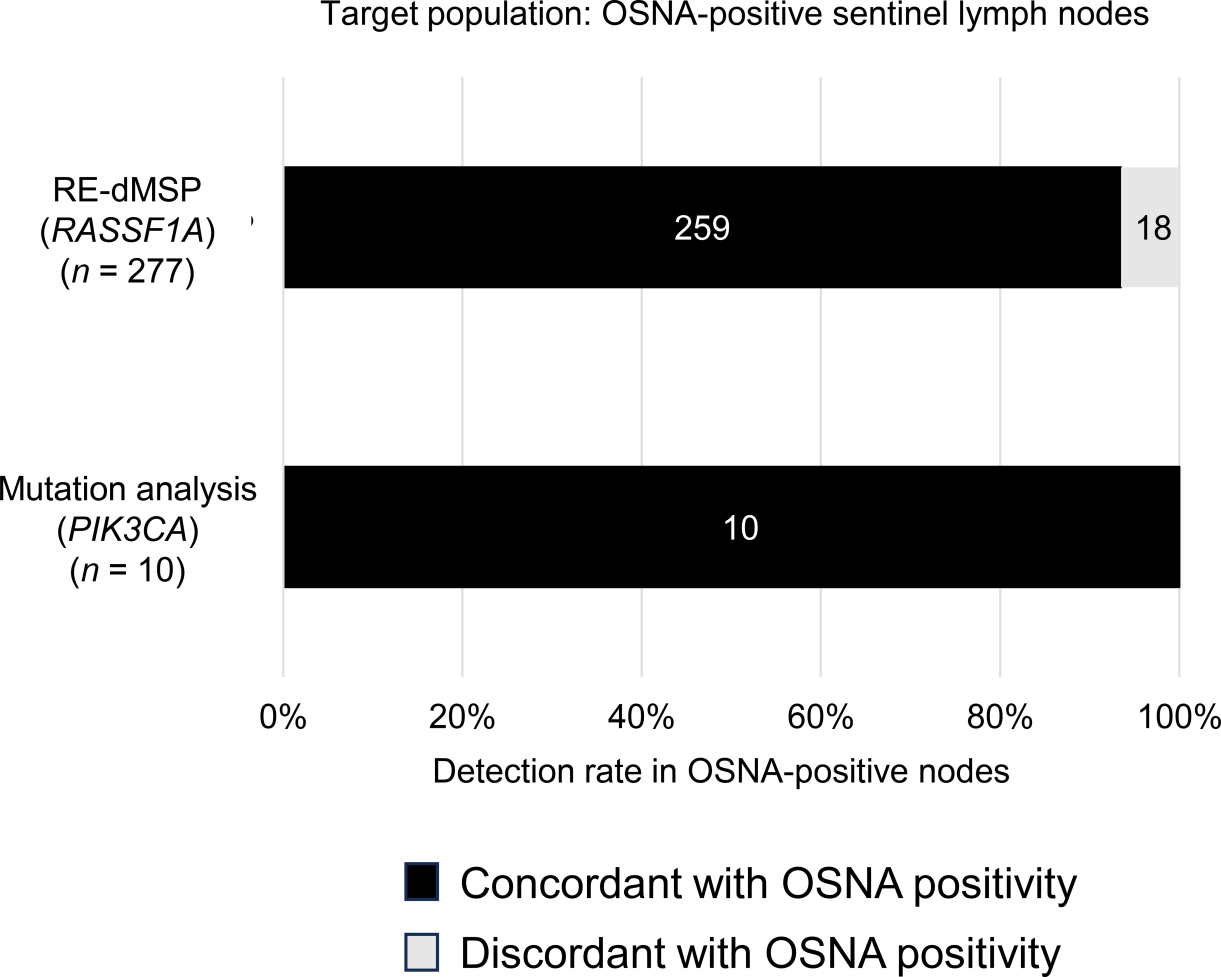
Detection rates for *RASSF1A* methylation (RE-dMSP) and *PIK3CA* mutation in one-step nucleic acid amplification (OSNA)-positive sentinel lymph nodes.

In the 59 SNs analyzed, a positive correlation was observed between the copy numbers of CK19 mRNA and mutated *PIK3CA* genes (*r* = 0.47, 95% confidence interval, 0.25–0.64, *p* < 0.01) (**Figure 4B**). This suggests that the *PIK3CA* mutation copy number might have potential as a clinical marker for evaluating tumor burden in SNs, as it showed a trend consistent with *CK19* mRNA levels. Of the cases of positive *PIK3CA* mutation in the primary tumor, it was possible to detect the *PIK3CA* mutation in all SNs assessed as metastatic by OSNA, with a 100% positive predictive rate for gene mutation detection (**Figure 5**).

## 4 Discussion

We have previously pointed out that DNA is more accurate than mRNA in assessments of the TTL of metastases in SNs (12), and discussed the usefulness of TTL assessment (10, 11). However, in practice, no search target has been identified that is more capable than CK19 mRNA of being used to comprehensively diagnose metastases in all breast cancers. Recent advances in genetic analysis technology have made comprehensive genetic analysis possible. The same applies to breast cancer treatment, and gene panel tests are widely used in clinical practice. In previous studies, it has been found that at least one case-specific genetic mutation is recognized in each case when whole-exome sequencing is performed (15). We focused on this report, analyzed case-specific mutated genes as a target for evaluation, and hypothesized whether the presence of these mutations in axillary lymph nodes could be assessed for TTL evaluation, postoperative adjuvant treatment, and treatment at recurrence.

In the present study, we specifically targeted *PIK3CA* mutations for ddPCR analysis primarily due to their high prevalence in breast cancer and direct clinical relevance as therapeutic targets. Furthermore, the concentration of these mutations in well-defined hotspots facilitates ddPCR assay design; this contrasts with other common mutations, which are often scattered across the gene. Given this context, we assessed whether these specific mutations could be detected in the axillary lymph nodes of breast cancer patients. We found that it was possible to identify the same mutated gene in all SNs assessed as positive for metastasis by OSNA in cases of a positive *PIK3CA* mutation in the primary tumor, with a positive predictive value of 100% (**Figure 5**). In our previous study on RE-dMSP, an assay for the diagnosis of axillary lymph node metastases for methylated *RASSF1A*, we observed cases in which lymph nodes assessed as positive for metastases by OSNA could not be assessed as positive by RE-dMSP, with a positive predictive rate of 93.5% (**Figure 5**). Thus, the results in the present study were better than those found in our RE-dMSP analyses. Although these results suggest that the current method may offer superior detection capability, this study is exploratory and hypothesis-generating. Furthermore, the clinical significance of ddPCR-positive/OSNA-negative cases remains to be elucidated, and we are currently investigating whether our findings reflect true metastatic potential. Identifying cases with true metastatic potential could enable the intensification of adjuvant therapy, thereby potentially preventing recurrence and improving prognosis.

Droplet digital PCR analysis of mutations in lysates, as conducted in the present study, requires knowledge of the mutational profile of the primary tumor. However, this information is not routinely assessed in clinical practice, and obtaining it requires time and resources for processes such as DNA extraction and primary site mutation analysis by next-generation sequencing. Therefore, ddPCR cannot, at present, be evaluated as a method of rapid intraoperative diagnosis. Nonetheless, the development of extraction and genetic analysis processes is expected to expand the use of ddPCR to intraoperative rather than postoperative diagnosis.

The limitations of the present study are as follows. First, even with highly sensitive DNA analysis, as used in the present study, prognostic differences have yet to be observed. Specifically, we found no clear correlation between the detection of mutations and clinical outcomes, such as recurrence, survival, or additional nodal disease. Consequently, the clinical significance of our results remains unclear. *PIK3CA* mutation–positive breast cancer has a poor prognosis (20–22); however, the reasons for this are not understood, and further studies with long-term follow-up are needed. Second, in the present study, only *PIK3CA* was analyzed as the target gene. We should investigate whether, by analyzing data from a greater number of cases, the results of the whole-genome sequence or whole-exome sequence could also be evaluated for other genes.

### 4.1 Conclusion

The absence of false negatives for OSNA in DNA analysis by ddPCR suggests the potential for more sensitive metastasis detection, although these findings are exploratory and hypothesis-generating. It is necessary to investigate whether, through further studies, the results of whole-genome and whole-exome sequencing could be evaluated for other genes.

## Data Availability

The original contributions presented in the study are included in the article/**Supplementary Material**. Further inquiries can be directed to the corresponding author.
