# Supplementary material for: Analysis of PIK3CA mutations in the lysate of sentinel lymph nodes in patients with early breast cancer
Source: Front Oncol. 2026 Mar 9;16:1658786. doi: 10.3389/fonc.2026.1658786 (PMC13006274; doi:10.3389/fonc.2026.1658786)
Supplement: Supplementary file 1 [file Table1.pdf]

Supplementary Table 1. Gene panel used for primary tumor sequencing and the number of cases in which each gene mutation was detected

Gene	Coverage, %	Cases of mutation detected (%)
PIK3CA	100	31 (33.0)
TP53	100	10 (10.6)
MAP3K1	100	9 (9.6)
GATA3	100	7 (7.4)
CDH1	100	6 (6.3)
PTEN	100	5 (5.3)
NCOR1	100	5 (5.3)
MAP2K4	100	3 (3.2)
AKT1	100	3 (3.2)
ERBB2	100	3 (3.2)
MUC1	97	2 (2.1)
RB1	100	1 (1.0)
ZNF217	100	1 (1.0)
CDKN2A	95	1 (1.0)
ESR1	92	1 (1.0)
CDKN2A	95	1 (1.0)
RPS6KB1	100	0
FGFR1	100	0
MDM4	97	0
MYC	100	0
CCND1	100	0
