# Supplementary material for: Analysis of PIK3CA mutations in the lysate of sentinel lymph nodes in patients with early breast cancer
Source: Front Oncol. 2026 Mar 9;16:1658786. doi: 10.3389/fonc.2026.1658786 (PMC13006274; doi:10.3389/fonc.2026.1658786)
Supplement: Supplementary file 2 [file Table2.pdf]

Supplementary Table 2. *PIK3CA* primers and probes used for droplet digital polymerase chain reaction in the present study

Gene primer or probe	Sequence (5′ to 3′)	Amplicon size, base pairs
H1047R		98
Forward primer	GCAAGAGGCTTTGGAGTATTTCATG	
Reverse primer	GCTGTTTAATTGTGTGGAAGATCCAA	
Probe (mutant)	FAM-CCACCATGATGTGCATC	
Probe (wild type)	VIC-CACCATGACGTGCATC	
E545K		81
Forward primer	TCAAAGCAATTTCTACACGAGATCCT	
Reverse primer	GCACTTACCTGTGACTCCATAGAAA	
Probe (mutant)	FAM-CTCTCTGAAATCACTGAGCAG	
Probe (wild type)	VIC-CTCTGAAATCACTAAGCAG	
E542K		90
Forward primer	CATTTTAGCACTTACCTGTGACTCCAT	
Reverse primer	GCTCAAAGCAATTTCTACACGAGAT	
Probe (mutant)	FAM-CTCTCTGAAATCACTGAGCAG	
Probe (wild type)	VIC-CTCTGAAATCACTAAGCAG	
