# Supplementary material for: Analysis of PIK3CA mutations in the lysate of sentinel lymph nodes in patients with early breast cancer
Source: Front Oncol. 2026 Mar 9;16:1658786. doi: 10.3389/fonc.2026.1658786 (PMC13006274; doi:10.3389/fonc.2026.1658786)
Supplement: Supplementary file 3 [file Table3.pdf]

Supplementary Table 3. Results of mutation analysis using data from the 94 patients in the present study, 31 of whom had *PIK3CA* mutations in the primary tumor

Mutated gene	*n*
PIK3CA hotspot	25
H1047R	13
E545K[a]	7
E542K	5
PIK3CA non-hotspot	7
H1047L	2
N345K	1
C420R	1
E674Q	1
E726K	1
H1048R[a]	1
Other	33
None	30

[a] One case had both *PIK3CA* E545K and H1048R mutations.
